# Patterns of attachment and reflective functioning in families of adolescents with eating disorders

**DOI:** 10.1186/2050-2974-2-S1-P10

**Published:** 2014-11-24

**Authors:** Elizabeth Seah, Lois Liley, Lynne Priddis, David Forbes, Jan Piek

**Affiliations:** 1 Specialised CAMHS Eating Disorders Program, Perth, Western Australia; 2 Curtin University, Perth, Australia; 3 University of Western Australia, Perth, Australia

## 

Eating disorders are serious, chronic disorders that are associated with significant physical, psychological and social costs. Many studies have demonstrated a connection between insecure patterns of attachment and eating disorders. In the last decade the theory of mentalization has emerged from the developmental and attachment theory literature in relation to mental health conditions. Mentalizing refers to a person's capacity to understand the thoughts, feelings, needs, intentions and desires that underlie the behaviour of self and others. The construct of mentalizing can be measured by a person's capacity for 'Reflective Functioning' (RF). Poor mentalizing capacity is seen to limit a person's ability to relate with others, maintain a sense of self and regulate difficult emotions. Deficits in mentalizing capacity have been linked to the psychopathology, including eating disorders.

This paper will present the current theory about the role of attachment theory and mentalizing in the development and maintenance of eating disorders. It will describe a study conducted to investigate attachment styles and mentalizing (reflective functioning) capacity of adolescents aged 13-17 with eating disorders, and reflective functioning of their mothers. The aims of the research project will be outlined, followed by details of the methodology. Finally, results including quantitative and qualitative data will be presented.

